# Research Status and Prospect on Vanadium-Based Catalysts for NH_3_-SCR Denitration

**DOI:** 10.3390/ma11091632

**Published:** 2018-09-06

**Authors:** Jie Zhang, Xiangcheng Li, Pingan Chen, Boquan Zhu

**Affiliations:** The State Key Laboratory of Refractories and Metallurgy, Wuhan University of Science and Technology, Wuhan 430081, China; zzzjwust@163.com

**Keywords:** vanadium-based catalysts, NH_3_-SCR, NO_x_, modification

## Abstract

Selective catalytic reduction of NO_x_ with NH_3_ is one of the most widely used technologies in denitration. Vanadium-based catalysts have been extensively studied for the deNO_x_ process. V_2_O_5_/WO_3_(MoO_3_)TiO_2_ as a commercial catalyst has excellent catalytic activity in the medium temperature range. However, it has usually faced several problems in practical industrial applications, including narrow windows of operation temperatures, and the deactivation of catalysts. The modification of vanadium-based catalysts will be the focus in future research. In this paper, the chemical composition of vanadium-based catalysts, catalytic mechanism, the broadening of the temperature range, and the improvement of erosion resistance are reviewed. Furthermore, the effects of four major systems of copper, iron, cerium and manganese on the modification of vanadium-based catalysts are introduced and analyzed. It is worth noting that the addition of modified elements as promoters has greatly improved the catalytic performance. They can enhance the surface acidity, which leads to the increasing adsorption capacity of NH_3_. Surface defects and oxygen vacancies have also been increased, resulting in more active sites. Finally, the future development of vanadium-based catalysts for denitration is prospected. It is indicated that the main purpose for the research of vanadium-based modification will help to obtain safe, environmentally friendly, efficient, and economical catalysts.

## 1. Introduction

Nitrogen oxides (NO_x_) are one of major air pollutants that could cause great harm to the ecological environment and human health. How to remove NO_x_ effectively is a significant issue of environmental protection. Currently, there are three kinds of reductant for the selective catalytic reduction of NO_x_: NH_3_, Urea and HC. NH_3_-SCR (selective catalytic reduction with ammonia) is the most mature industrial technology at present, and its main reaction process [1,2,3] is:

4NH_3_ + 4NO + O_2_ → 4N_2_ + 6H_2_O (standard SCR reaction) (1)

4NH_3_ + 2NO + 2NO_2_ → 4N_2_ + 6H_2_O (fast SCR reaction) (2)

The technology has been used in hundreds of large power plants in the United States, Germany, and Japan. Environmental conditions have a great impact on this technology. For example, it is important to control oxygen content of the exhaust appropriately for NH_3_-SCR. In the absence of O_2_, the reducing agent cannot reduce NO. When the oxygen content of the exhaust is below 5%, the catalytic reduction reaction of NH_3_ can be carried out to convert NO_x_ into N_2_ and H_2_O. When the oxygen concentration is too high, the activity of the catalyst will be reduced, and NH_3_ will be oxidized to NO_2_, resulting in new pollution.

The catalyst is the key to determining the reactivity of NH_3_-SCR under the proper external conditions. In the 1960s, a study on exhaust gas-purifying catalysts was started. A three-way catalyst (TWC) consisting of precious metals was researched in the initial stage. Although the catalytic effect is ideal, considering the cost and the source of precious metals, the direction has shifted to transition metal oxide catalysts. Vanadium-based catalysts are used due to the high activity of SCR reactions. With the continuous improvement of environmental protection and energy awareness and the increasing demand for low NO_x_ emissions, the traditional vanadium-based catalysts are no longer satisfactory. The performance improvement of vanadium-based catalysts has become the focus of research.

In this paper, the characteristics and performance improvement of vanadium-based catalysts is the main line. The research progress of vanadium-based catalysts at home and abroad is reviewed. The advantages and disadvantages, mechanism, and improvement methods are summarized. The development of vanadium-based catalysts for denitrification is prospected.

## 2. Composition of Vanadium Based Catalyst

The catalyst is obtained in such a way that the active component and promoter are loaded on catalyst support. The catalyst support is believed to be important for SCR reaction because of its high surface areas, good thermal stability, and high active substance dispersion on surfaces [4]. In general, TiO_2_–anatase is widely used as a support for vanadium-based catalyst because of its great SO_2_ resistance and excellent dispersion of V_2_O_5_. A small amount of sulfate species loaded on the support enhances the surface acidity of the V_2_O_5_/TiO_2_ catalyst, increases the amount of adsorbed NH_3_, and improves the SCR activity [5,6]. However, pure TiO_2_ is poorly active during the SCR reaction due to its weak acidity and poor redox ability. Additionally, TiO_2_ will transform from anatase to rutile at high temperatures, which will affect the stability of the support. Therefore, some modifications have been investigated to overcome these defects. Adrian, M. et al. [7] chose TiO_2_-WO_3_-SiO_2_ as a catalyst support for optimization, using rare metal to inhibit the phase transition of titanium dioxide, to avoid high temperature sintering and surface area loss, and improve the structural strength of the support. At the same time, SiO_2_ is beneficial to SO_2_ resistance. Cheng, J. et al. [8] synthesized TiO_2_-PILC as a catalyst support for the SCR of NO_x_ with NH_3_ due to pillared interlayered clays’ large specific surface areas, acidity, and high thermal stability. Other catalyst support is also helpful for the performance of the catalyst, such as TiO_2_-pal [4], and activated carbon (AC) [9].

**Figure 1:**
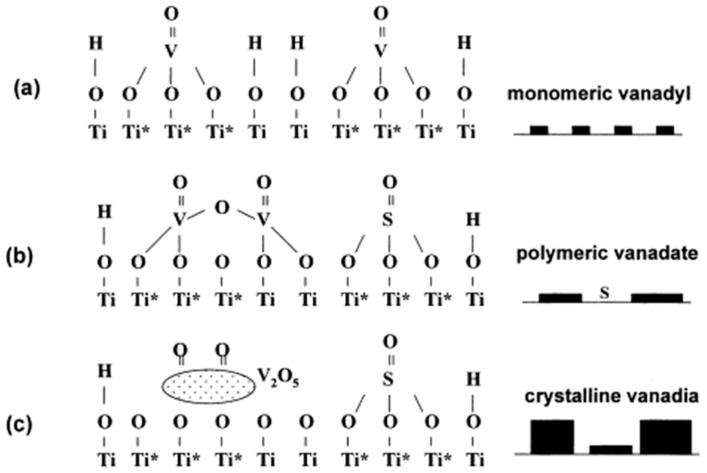
A schematic of the transformation of V_2_O_5_ on titania: (**a**) low loading V_2_O_5_ supported on sulfur-free TiO_2_; (**b**) low loading V_2_O_5_ supported on sulfated TiO_2_ and (**c**) high loading V_2_O_5_ supported on sulfated TiO_2_ (Ti*, basic site; Ti, neutral site). Reproduced with permission [20]. Copyright 2000, Elsevier.

The active component is the dominant factor affecting vanadium-based catalyst performance, and it can be divided into single vanadium oxide VO_x_ [10,11] and multi-metal oxide compounds such as VO_x_-WO_3_ [12], VO_x_-CeO_x_ [8]. V_2_O_5_ catalyst is a kind of structure-sensitive catalyst, whose activity is affected by the structure and coverage of V_2_O_5_, the crystal form of catalyst support, and the modification element. Increasing the amount of V_2_O_5_ added can help to improve the catalytic activity. However, V_2_O_5_ will promote the transformation of anatase to rutile, which is not conducive to the thermal stability of the catalyst, resulting in the shortened life of the catalyst and the decrease of selectivity [13]. In view of this, the content of vanadium in the commercial catalyst VO_x_-WO_x_/TiO_2_ is generally limited to less than 2.6%. The coverage rate is also an important parameter, which can be adjusted by the V loading or the calcination temperature [14]. The coverage rate for ideal V_2_O_5_-WO_3_/TiO_2_ catalysts is 25–50%. Below 25% indicates that the vanadium content is too low or the surface area is too high and the catalyst activity is low. With coverage of more than 50%, the stability of the catalyst and the conversion of NO_x_ at high temperatures cannot be guaranteed [7,14]. The vanadium coverage of conventional catalysts is below the dispersion limit that monolayer 6–7 atom /nm^2^ to avoid the crystalline V_2_O_5_ [15]. It is generally believed that in the dry anatase surface at low loading V_2_O_5_, the dehydrated surface vanadium generally tend to possess isolated four-fold coordination. The isolated VO_4_ units consist of one terminal V=O bond and three bridging V-O-Ti bonds, as shown in Figure 1a. As the coverage increases, the number of V-O-V bridges per V increases. In practical application, the vanadium species mainly exists in the form of polyvanadate, as shown in Figure 1b. This species has higher SCR reactivity than the monomer (Ti-O)_3_V=O [3,15,16]. At high loading V_2_O_5_, crystalline vanadia is generated on sulfated TiO_2_ (Figure 1c). In view of the volatilization of vanadium at high temperatures, the use of metal vanadate (MeVO_4_) in the active phase can increase the melting point of the catalyst [17,18]. Moreover, Zhou, X. et al. [19] synthesize the V@Mn-Fe/ATP catalyst with a similar core-shell structure successfully. It was found that the V_2_O_5_ layer as a coating might protect other active metals of the catalyst.

## 3. NH_3_-SCR Reaction Mechanism of Vanadium-Based Catalyst

**Figure 2:**
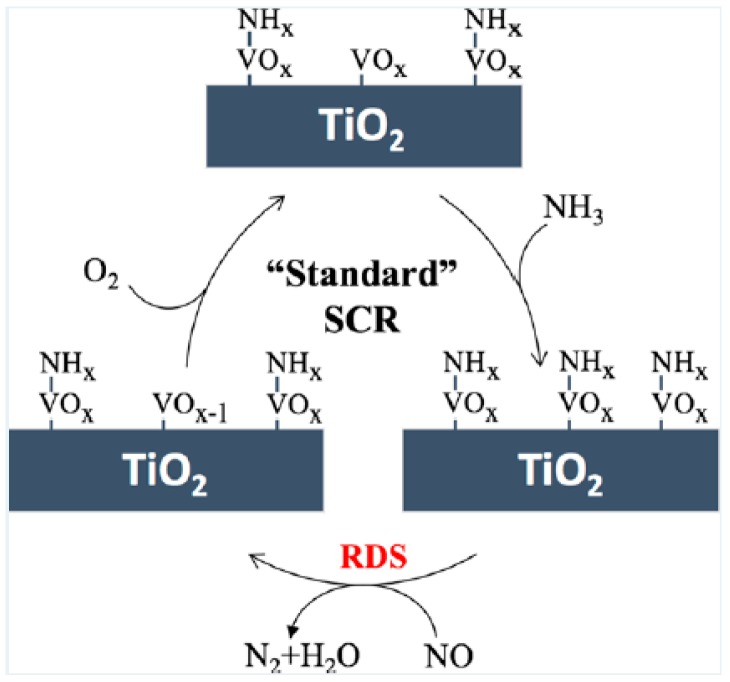
Schematic diagram of the standard SCR reaction. Reproduced with permission [2]. Copyright 2017, American Chemical Society.

Zhu, M. et al. [2] used elemental tagging to study the SCR reaction pathway of vanadium-based catalysis and found that the SCR reaction occurs at the surface V^5+^O_4_ site. This is shown in Figure 2, which exhibits a schematic diagram of the standard SCR reaction and indicates that SCR reaction is divided into three steps. First, NH_3_ adsorption is observed on the surface of the acid center. Next, the adsorbed ammonia reacts with NO to form an intermedium. The intermedium can decompose to N_2_ and H_2_O and reduce surface V^5+^O_4_ sites. Finally, the reduced V^4+^O_3_ is reoxidized by O_2_. Through the kinetic study, the rate-determining step of the SCR reaction is that the V^5+^ on the surface is reduced to V^4+^ by NO and NH_3_, particularly the breaking of N–H bonds during the process of formation or decomposition of the intermedium. The vanadium-based catalyst V_2_O_5_-WO_3_/TiO_2_ has been commercialized and applied in some thermal power plants. In V_2_O_5_-WO_3_/TiO_2_ catalysts, the O=VO_3_ site is involved in the redox cycle, which is the most important for the SCR reaction. The O=WO_4_ site enhances the surface acidity of the catalyst and promotes the adsorption of NH_3_ [12]. Tungsten oxide as a promoter has two primary roles in catalysis: it restrains the generation of bulk polymeric V aggregates and retains Brønsted acid sites at high temperatures [21].

**Figure 3:**
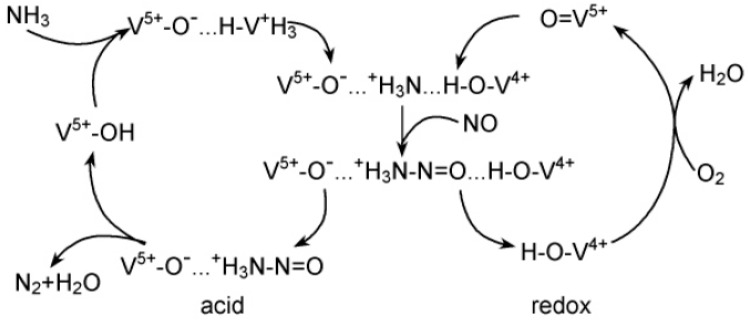
Schematic diagram of NH_3_-SCR reaction mechanism. Reproduced with permission [22]. Copyright 1995, Elsevier.

Although vanadium-based catalysts have been used in industrial applications, the reaction mechanism for vanadium-based catalysts remains controversial. It is mainly divided into two theories: the Langmuri-Hinshelwood theory (L-H theory) and the Eley-Rideal theory (E-R theory). It is widely believed that the vanadium-based catalyst selectively reduces NO_x_ via the E-R mechanism at medium-high temperature. A schematic diagram of the NH_3_-SCR reaction mechanism is shown in Figure 3. Although ammonia is adsorbed on both Lewis acid sites and Brønsted acid sites, NH_3_ is more prone to be adsorbed at the Brønsted acid sites than at the Lewis acid sites. NH_3_ forms NH^4+^ on the Brønsted acid site (V–OH) and reacts with the V=O group to generate intermediate products, and then decomposes into N_2_ and H_2_O. However, NH_3_ adsorption on Lewis acid sites will produce N_2_O [3].

Aside from the Langmuri-Hinshelwood model and the Eley-Rideal model, a surface Mar-van Krevelen reaction mechanism has been proposed for the SCR reaction. According to the Mar-Van Krevelen mechanism, SCR reaction divides into three steps: adsorption, migration, and reoxidation. There are two different types of centers that prevail on the SCR catalysts: S1 sites, related to vanadium and its redox cycle, and S2 sites, used to store ammonia and nitrates [23]. However, there is controversy around the step of vanadium reoxidation. According to the sites of detached gaseous oxygen, two different possibilities for vanadium reoxidation steps were proposed: in one case that the vacancy in the vanadia lattice is filled using molecular oxygen, and the other through the creation of a vacancy in the titania lattice [24].

In fact, the SCR reaction mechanism is closely related to the reaction temperature. With different reaction temperatures, the number and distribution of the active sites of the catalysts are different, and the types, quantities and distributions of the acids are not the same. Therefore, the reaction mechanism of vanadium-based catalysts may also change with different reaction temperatures. It is worth noting that current studies only focus on the formation of acid centers and the activity of acids in acid-base mechanism. It also has a certain significance to study the effect of alkaline position on the adsorption and the activity [3].

## 4. Performance Improvement of Vanadium Based Catalysts

**Figure 4:**
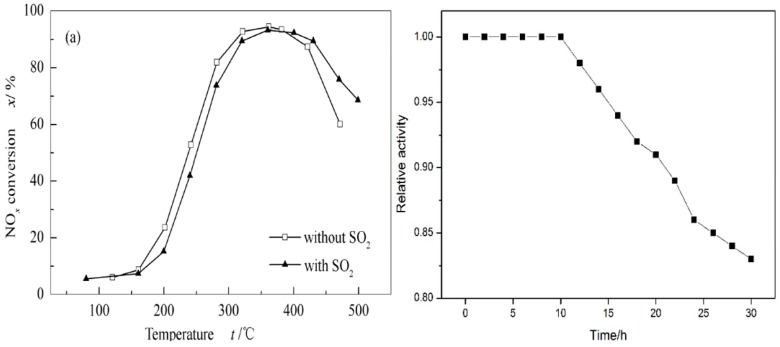
(**a**) NO_x_ conversion rate under different conditions [17]; (**b**) The relative activity of SO_2_ varies with time.

Commercial vanadium-based catalysts are widely used in industry due to their high activity and excellent SO_2_ resistance. However, they have a narrow activity window, and weak ability for resisting VO_X_ species to thermal sintering. The metal oxide is also over-oxidized, which not only oxidizes NH_3_ to N_2_O and NO, but also oxidizes SO_2_ to SO_3_. The generated SO_3_ continues to react with NH_3_ to generate ammonium sulfate species, thereby plugging the pores and masking the active sites, thus decreasing the activity of the SCR reaction [25]. The inevitable soot and some alkali metals in factory waste can also affect the activity of catalysts and even lead to deactivation of catalysts [4,5,26]. The evaluation of vanadium-based catalytic activity is shown in Figure 4. It can be seen that the NO_x_ conversion rate of conventional catalysts is higher than 90% at 300–400 °C, and has little effect in the presence of SO_2_. However, through experiments, we found that the longer the SO_2_ exists, the greater the decrease of catalyst activity as shown in Figure 4b. In this case, the performance improvement of vanadium-based catalysts has been the focus of research. In order to improve the activity of vanadium-based catalysts, the method usually has been adopted by changing the catalyst preparation, improving the performance of the support, adding modification elements and so on.

Usually the preparation of catalysts requires drying and calcining, and other processes. Some catalysts are activated by calcination. The calcination temperature affects the crystal structure of the catalyst. The adsorption sites and the adsorption numbers of different crystal planes are different. The surface sites’ variation lie with the nature of the oxide, preparative method used, conditions of activation, and impurities [27]. At the same time, both lattice defects and inhomogeneous surfaces have an effect on the catalytic properties.

The crystal form of catalyst support has a significant influence on SCR reactions, for example, TiO_2_-anatase has higher dispersibility to active components than TiO_2_-rutile and leads to greater performance. The methods of preparing catalyst supports include impregnation, coprecipitation, hydrothermal, and sol-gel. The impregnation method is the most widely used method because of its simple and easily-operated process. These methods mainly control the particle size and morphology of the support, as well as the binding strength of active components to the support, thus affecting the dispersion of active components and the activity of the catalyst. Pappas, D.K. et al. [28] have used the alkaline hydrothermal method to prepare titanium nanotubes and co-precipitation to prepare spherical nanoparticles and nanotubes for comparison. It was found that different forms of supports have certain effects on the SCR reaction. Using titanium nanotubes as support has a better effect than spherical titanium nanoparticles. This is because the multilayer structure of the titanium nanotube can provide greater surface area to facilitate the dispersion of active components, so as to provide more active sites. Vuong, T.H. et al. prepared CeO_2_ supports by a citrate or a precipitation method and then got loading V/CeO_2_ catalysts by wet impregnation. Comparing with the citrate sol-gel protocol, the mesoporous CeO_2_ support prepared by the precipitation method is much more active due to markedly higher surface area, higher concentration of Ce^3+^ bulk species and oxygen vacancies, which are beneficial for oxygen transport through the lattice [10].

Adding a modified element to modify the catalyst is beneficial to improve the performance of vanadium-based catalysts. Different modified elements may cause different effects on the VO_x_ active sites because of different physical and chemical properties of the surface. The presence of surface acid sites depends on both the specific oxide-supported ligand and the surface density of the metal oxide overlayer [29]. At ambient conditions, vanadium species on titania has distorted the octahedral oxygen environment with one short (V=O) and one long V–O axial bond at low loading V_2_O_5_ [30]. The theoretical value for monolayer coverage of dehydrated VO_x_/TiO_2_ catalysts is 7.9 atom/nm^2^ [29]. Due to stronger interactions between vanadium and ceria, vanadium oxide species disperse on ceria up to 9 V atom/nm^2^ of support determined by Raman Spectroscopy [31]. Vanadia reacts with cerium oxide to form CeVO_4_ phase, so two types of vanadium species (VO_x_ and CeVO_4_) are dispersed on the surface of cerium dioxide. CeVO_4_ promotes the formation of Brønsted acid sites while VO_x_ and CeO_2_ provide Lewis acid sites on the surface [32]. However, the formation of Brønsted acid sites is related to vanadium species VO_x_ in bulk CeVO_4_ after aging at 500 °C [18]. Therefore, the Lewis acid sites may be converted to the Brønsted acid sites at high temperature SCR reaction. In bulk CeVO_4_ catalysts, the isolated vanadium species in the tetrahedral coordination are basically stable after the zircon-type structure CeVO_4_ aging at 500 °C [18]. In the Ce-doped V_2_O_5_-WO_3_/TiO_2_ catalyst, the presence of Ce^3+^ creates a charge imbalance, vacancies, and unsaturated chemical bonds, thus increasing the chemisorbed oxygen on the surface. In addition, the coexistence of Ce^4+^ was found to play an important role in the synergic effect on the SCR reaction [33]. At ambient conditions for fresh Fe-V/TiO_2_ catalysts, the structures of the vanadium species exist in octahedral (70%) and tetrahedral (30%) coordination [34]. The acid sites on V_2_O_5_-Fe_2_O_3_/TiO_2_ are mainly derived from Fe_2_O_3_-TiO_2_, so the adsorbed NH_3_ prefer to be activated by Fe^3+^, and then V^5+^ accelerate the regeneration of Fe^3+^ [35]. In FeVO_4_/TiO_2_ catalysts, the FeVO_4_ loading amount should be less than 9 wt% to avoid exceeding the monolayer dispersion limit. The Fe^3+^-O-V^5+^ linkages with a low coordination number of vanadium atoms are associated with structural defects on the surface. The presence of an electronic inductive effect between Fe^3+^ and V^5+^ was beneficial for high deNO_x_ efficiency and N_2_ selectivity [36]. At low temperatures, FeVO_4_ shows low activity for SCR in FeVO_4_/TiO_2_-WO_3_-SiO_2_ catalysts. However, in the decomposition of FeVO_4_, the VO_x_ species exhibits higher SCR activity. So, the active species for SCR is not FeVO_4_ but VO_x_ species [37]. The presence of iron can be useful in altering the electron density of the VO_x_ surface species and inducing more Lewis acid sites and Brønsted acid sites for SCR reaction [38]. Currently, in the catalytic denitrification reaction of manganese-based bimetallic oxide catalysts, Mn-OH as a Brønsted acid site adsorbs NH_3_ to form NH_4_^+^. NH_4_^+^ is then reacted with NO to create N_2_, H_2_O and Mn^3+^–OH. Finally, Mn^3+^ is reoxidized to regenerate Mn^4+^ [39]. The addition of Mn shows a promoting effect on the activity of the V_2_O_5_/TiO_2_ catalyst because manganese oxides contain various valence states, facilitating the completing of the catalytic cycle. As promoter, the ideal loading of Mn is about 2% for V_2_O_5_/TiO_2_ catalysts to avoid the oxidation of NH_3_ at high temperatures. The presence of the synergetic effect between V and Mn contributes the improvement of SCR activity. The redox cycle (V^4+^ + Mn^4+^ ↔ V^5+^ + Mn^3+^) reduces the energy required for electron transfer between Mn and V active sites, and promotes the adsorption and activation of NH_3_ and NO. Mn^3+^ is conducive to NH_3_-SCR reactions due to its ability to induce the formation of more surface chemisorbed oxygen [40]. In Cu-V/TiO_2_ catalysts, the copper species exist mainly as Cu^2+^ in CuV_2_O_6_, Cu_2_V_2_O_7_ or CuO. Different bimetallic copper vanadate oxides have different configurations, for example, distorted octahedral VO_6_ sub-units and tetrahedral VO_4_ sub-units. VO_4_ units are more convenient to access to NH_3_ due to their open V environment. By introducing copper species, strong acid sites are increased. It is worth noting that Lewis acid sites are the strong acid sites in Cu–V/TiO_2_ catalyst [21,41].

The performance improvement of vanadium-based catalysts is mainly in the following two directions. The first is to widen the temperature window of vanadium-based catalysis, so that it can guarantee the activity of the medium-temperature section and have very good low-temperature activity at the same time. The second is to improve the resistance to sulfate poisoning of vanadium-based catalysts and the erosion ability of water and alkaline metals. Many researchers adopted different methods of improvement and achieved varying results.

### 4.1. The Broadening of the Temperature Ranges

In practice, the temperature of exhaust gas is about 200 °C. Therefore, the broadening of the temperature range is mainly important for the low temperature section. Adding modified elements (Cu, Ce, Fe, Mn, etc.) is a more effective method to solve it.

**Figure 5:**
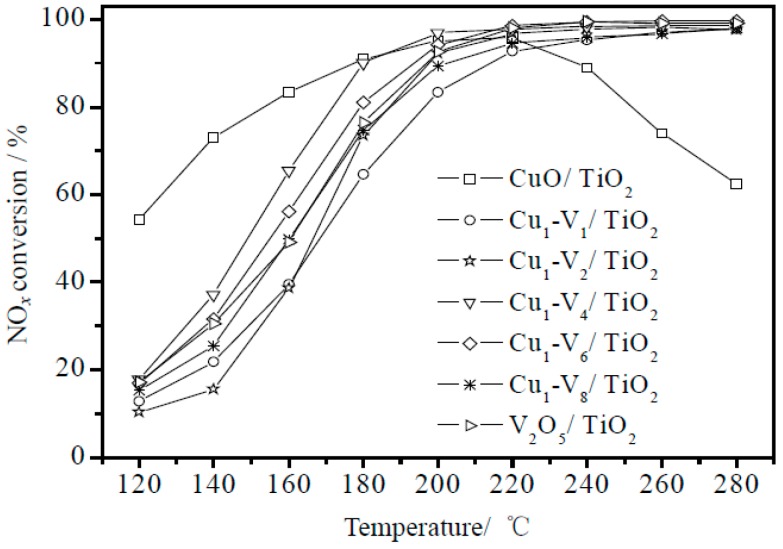
Conversion rate of NO_x_ at different reaction temperatures [6].

The Cu based catalyst has good low temperature activity. It has been found that the combination of copper oxide and other metal oxides can obtain better activity. The NO_x_ conversion rate of the catalyst for the Cu–V system is described in Figure 5. It can be seen from diagram that at the optimum Cu–V ratio, the conversion of NO_x_ reaches 90% at 180 °C and nearly 100% at 220 °C [6]. The synergistic effect of Cu and V alleviated the influence of VO_x_ aggregation on TiO_2_ crystal form, enhanced the adsorption capacity of the catalyst surface, and facilitated to the SCR reaction at low temperatures. However, Cu is conducive to forming CuSO_4_ with SO_2_, and the ability of resisting SO_2_ poisoning is poor [5,26].

**Figure 6:**
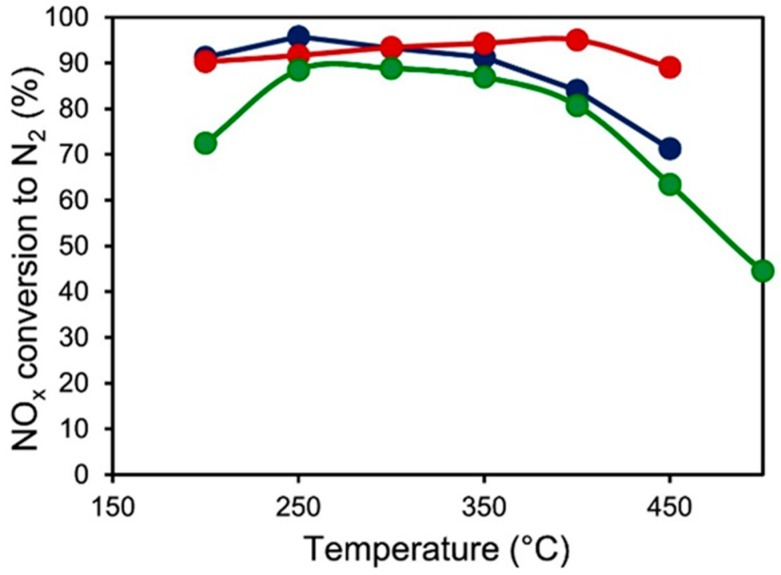
The activity evaluation of the catalyst at different calcining temperatures: after aging at 500 °C (full symbol in red); after aging at 600 °C (full symbol in green); fresh (full symbol in blue). Reproduced with permission [18]. Copyright 2017, Elsevier.

Ce, as a rare earth element, has been studied more in recent years. It has some kind of role of "backup chemical bond" due to the variability of the coordination number. As a modified element, it could facilitate the valence of vanadium species rapidly changing by a redox cycle of V^5+^ + Ce^3+^ ↔ V^4+^ + Ce^4+^. There are experiments using CeVO_4_ as a precursor loaded onto the support and compared with the performance of V_2_O_5_/TiO_2_ [7,18]. It shows that the catalyst has a high catalytic activity and a wide temperature window with the best Ce content and a suitable calcination temperature (vanadate decomposition temperature). The activity evaluation of the catalyst with different calcining temperatures in fast conditions is shown in Figure 6. It exhibits that the NO_x_ conversion rate reaches over 90% at the optimum heat treatment temperature, from 200–450 °C. The calcined CeVO_4_ exhibited better NO conversion and N_2_ selectivity. The redox ability of Ce can increase the surface acidity of the catalyst and improve the storage capacity of oxygen. Ce^4+^/Ce^3+^ can form solid solutions with other oxides to increase the active sites. Meanwhile, Ce can effectively reduce the sintering tendency [7,42]. In addition, the activity distribution of V_2_O_5_/CeO_2_ mixed oxides is somewhat complex. The existence of CeVO_4_ and the correlation of the V–O–Ce subsequently constitute two systems: VO_x_/CeO_2_ and CeVO_4_/CeO_2_. The coexistence of low concentration of Ce^4+^ species assists to enhance the rate NO conversion to nitrogen [18]. And the synergistic effect of two oxides of CeO_2_ and V_2_O_5_ generated by the decomposition of cerium vanadate promotes redox cycle and improves the activity of the catalyst.

**Figure 7:**
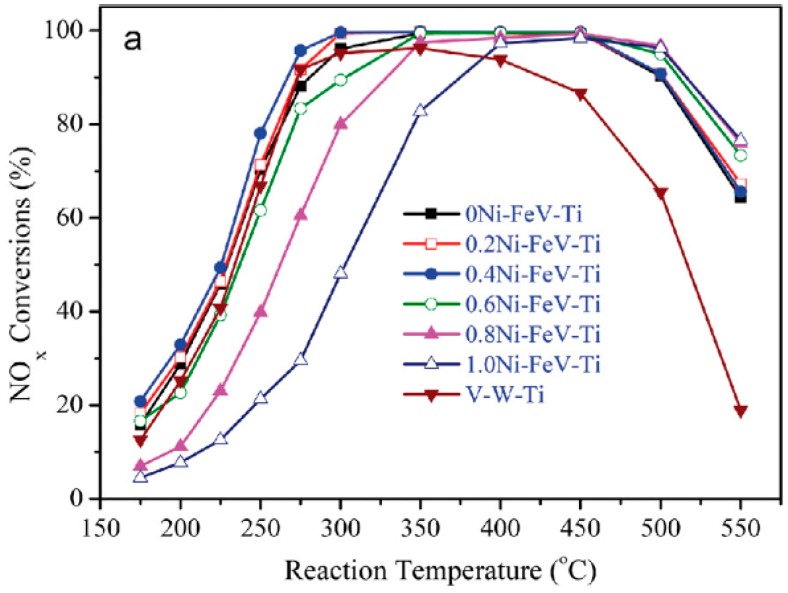
Catalytic activity evaluation of different catalysts in Fe–Ni–V system. Reproduced with permission [44]. Copyright 2017, Elsevier.

Iron-based catalysts have attracted much attention because of their cheap, nontoxic, and high hydrothermal stability and N_2_ selectivity. But at low temperature, they exhibits a poor conversion rate of NO_x_. In SCR reactions temperatures below 300 °C, the active site of iron is mainly in the form of isolated Fe^3+^. When the temperature rises, oligomeric iron oxide clusters, and iron oxide particles become dominant. This contributes more to the SCR reaction and makes it more active. The introduction of Fe can increase the weak acid sites of the catalyst and the NH^4+^ formed by NH_3_ in the weak acid sites is more easily attached and activated in the SCR reaction [17]. Nickel added as a modified element could change the electronic environment of the metal surface through the formation of heteroatom bonds, thereby modifying its electronic structure with the ligand effect, improving the surface properties [43]. Wu, G. et al. [44] have conducted experiments by adding nickel to the FeV/Ti catalyst to investigate the effect on the SCR reaction. Figure 7 shows the evaluation of the catalytic activity of different catalysts in the Fe–Ni–V system. It can be seen that, with the optimum proportion, the catalytic activity of NO_x_ reaches over 90% at 250–550 °C, and the NO_x_ conversion rate at 280–500 °C reaches 100%. The results show that Ni promotes the interaction between FeVO_4_ nanoparticles and TiO_2_, and improves the acidity and reducing activity. When the content of Ni is 0–0.4, Ni is highly dispersed on the surface of FeVO_4_. When the content is 0.6–1, Ni reacts with FeVO_4_ to form nickel-iron spinel, so that more defects and oxygen vacancies are formed on the surface of the catalyst. The addition of Ni affects the grain size, changes the surface properties of the catalyst, has better activity at the test temperature, and can widen the temperature window.

**Figure 8:**
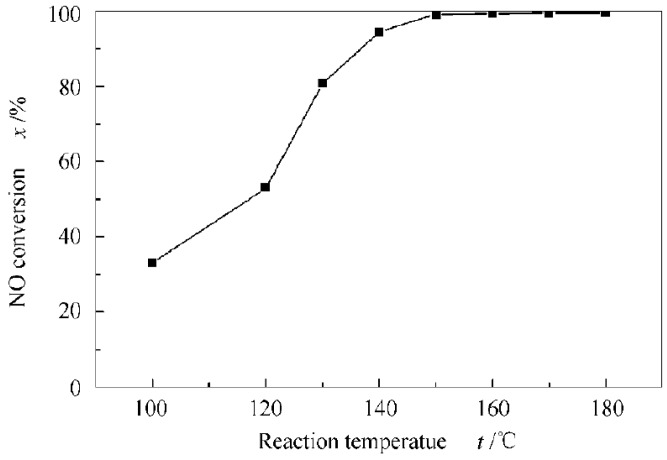
Activity evaluation of Mn-V-Ce/TiO_2_ catalysts at different temperatures [47].

Manganese bases have attracted attention due to their high activity at low temperatures. The crystalline phase structure of the manganese-based catalysts prepared by different methods can affect the reactivity of SCR, and the irregular surface structure causes more defects and active sites, thus increasing the reactivity of SCR. Liu, J. et al. [45] have studied the SCR reaction mechanism of manganese-based catalysts and believe that the highly active manganese reacts mainly according to the L–H reaction mechanism at low temperature. Pure MnO_x_ is unsuitable as a catalyst because of its low surface area and poor thermal stability [46]. However, manganese-based catalysts have lower N_2_ selectivity, and are easily deactivated under SO_2_ and H_2_O conditions, causing irreversible loss of oxidation ability, resulting in the blockage of pores and active sites by ammonium sulfate salts, limiting the further popularization. Therefore, Zheng, Z. et al. [47] utilize the excellent low-temperature performance of MnO_x_ to make up for the shortcoming of the narrow temperature window of V_2_O_5_/TiO_2_ and prepare an Mn–V–Ce/TiO_2_ catalyst. This catalyst uses Mn to provide low-temperature active sites, and Ce and V provide sufficient oxygen vacancies while enhancing the surface acidity, thereby significantly improving the low-temperature activity. As shown in Figure 8, the activity of Mn–V–Ce/TiO_2_ catalyst at different temperatures is evaluated. It is found that the NO_x_ conversion rate is as high as 99.2% at 150 °C, indicating excellent low-temperature activity of the catalyst.

### 4.2. The Improvement of Erosion Resistance

V_2_O_5_-WO_3_(MoO_3_)/TiO_2_, which has been put into industrial use, is subjected to a lot of erosion in the actual industry environment, one of which is high concentrations of SO_2_. V_2_O_5_ possesses SO_2_ durability, but SO_3_ is generated due to peroxidation, and the resulting sulfuric acid (hydrogen) salt will affect the catalyst activity. The addition of WO_3_ is helpful to increase the Brønsted acid sites, enhance the reaction activity by improving the redox capacity at low temperatures, and to restrain the phase transition, but it still faces erosion of hydrogen sulfate. Li, C. et al. [48] studied the formation and decomposition of ammonium bisulfate over V_2_O_5_-WO_3_/TiO_2_ catalysts at different temperatures. With the combination of ammonium hydrogen sulfate (ABS) and metal oxides (WO_3_ and TiO_2_), electrons deviate from the sulfate, thereby weakening the stability of the ABS and reducing the decomposition temperature. However, the vanadium-generated intermediate VOSO_4_ makes the sulphate electrons saturated. The presence of NO and O_2_ could break the bonds inside the ABS, and react with the ammonia species produced in it, allowing the NH_3_ to be separated from the ABS, accelerating the decomposition of the ABS and competing to inhibit its formation. It was found that the trace ABS on TiO_2_ had little effect on the NH_3_-SCR reaction at 300 °C. Under 300 °C, sulfuric acid (hydrogen) ammonia species cover the active sites, masking VOSO_4_ intermediate products, resulting in a poor NH_3_-SCR reaction performance. Therefore, it is necessary to study the SO_2_ corrosion resistance of catalysts applied to low temperature.

**Figure 9:**
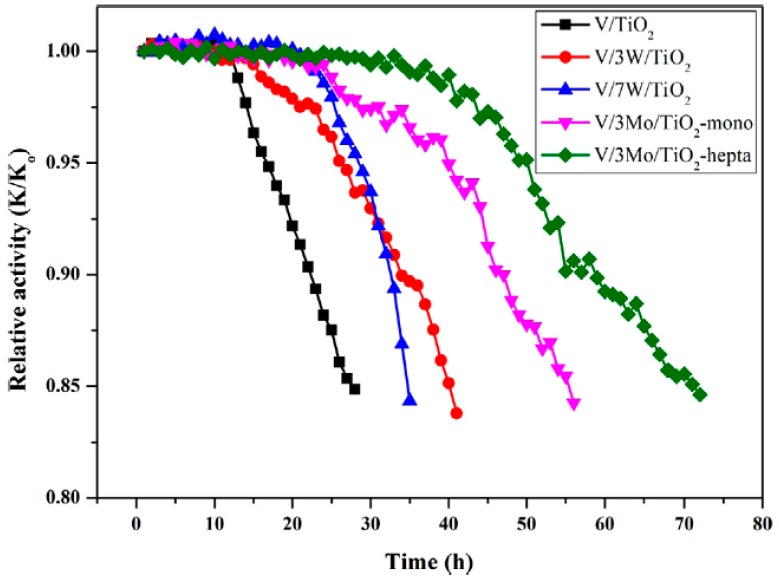
Relative activity in the presence of SO_2_ for the SCR of NO by NH_3_ over V/TiO_2_, V/3W/TiO_2_, V–3Mo/TiO_2_-mono and V/3Mo/TiO_2_-hepta at 250 °C. Reproduced with permission [49]. Copyright 2016, Elsevier.

Compared with WO_3_, MoO_3_ is less affected by SO_2_ and the chemical properties of MoO_3_ are similar to those of WO_3_. Kwon, D.W. et al. [49] have used ammonium molybdate (NH_4_)_2_MoO_4_ and ammonium heptamolybdate (NH_4_)_2_Mo_2_O_7_ as precursors of molybdenum sources, and ammonium metatungstate hydrates as precursors for tungsten sources, compared with WO_3_ and MoO_3_ properties, to study MoO_3_’s ability of resisting SO_2_. The catalyst was characterized by X-ray photoelectron spectroscopy (XPS) analysis, and the results showed that the binding energy of ammonium heptamolybdate was higher than that of ammonium molybdate. High binding energy can promote the interaction of metal atoms and oxygen, which is beneficial to the redox and SCR reactions of NO_x_. Furthermore, the Mo^6+^/Mo^5+^ of ammonium heptamolybdate is higher than that of ammonium molybdate. The presence of Mo^6+^ on the surface of the catalyst can inhibit the reaction between SO_2_ and V=O, reduce the absorption of SO_2_, and thus play the role of resisting SO_2_. Therefore, high Mo^6+^/Mo^5+^ exhibits excellent resistance to SO_2_. Figure 9 shows relative activity in the presence of SO_2_ for the SCR of NO by NH_3_ over different catalysts at 250 °C. It can be seen that the SO_2_ has a certain effect on the catalyst. However, compared to the V_2_O_5_/TiO_2_ and the V_2_O_5_/WO_3_ system, the time for V_2_O_5_/MoO_3_ to remain active is more prolonged. That is, V_2_O_5_/MoO_3_ is more resistant to SO_2_.

**Figure 10:**
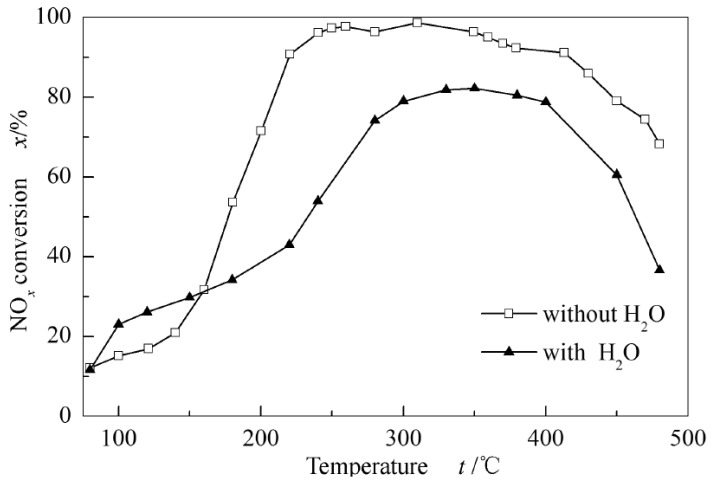
The effect of water vapor on NO_x_ conversion of Fe_1_V_1_/TiO_2_ catalyst [17].

It is important to note that, when testing against SO_2_ toxicity, the controlled erosion conditions are only SO_2_. However, in practical applications, the emitted gas may contain a certain amount of water vapor. At this point, SO_2_ and H_2_O, NH_3_ more easily form ammonium sulfate on the surface of the catalyst to deactivate the catalyst. Generally, at temperatures between 200 and 300 °C, vanadium-based catalyst SCR reactions are strongly affected by coordination of water. The adsorbed ammonia on oxo vanadia Lewis acid sites and ammonium ions associated with Brønsted acid sites selective reduce weakly adsorbed NO via the E-R mechanism, and the water appears to shift the equilibrium between NH_3_ and NH_4_^+^. At this time, the ratio between Lewis acid, Brønsted acid sites and V=O sites depend on the content of water vapor [15]. Because of the low SO_2_ resistance of V_2_O_5_-WO_3_/TiO_2_, there are experiments showing that selecting Ce to replace V and the prepared Ce_0.2_W_0.2_TiOx has good NOx conversion activity and N_2_ selectivity [42]. The activity of the catalyst was stable under the condition of SO_2_ only, while the decrease of the activity of the catalyst was observed under the condition of both SO_2_ and H_2_O. In addition, the performance of the Fe_1_V_1_/TiO_2_ catalyst was tested with an aqueous test and anhydrous test, as shown in Figure 10, and the results showed that the activity in the anhydrous test was obviously better than that of the water test [17]. Based on this, it is necessary to discharge the prior drying or improve the resistance of the catalyst to water erosion performance.

**Figure 11:**
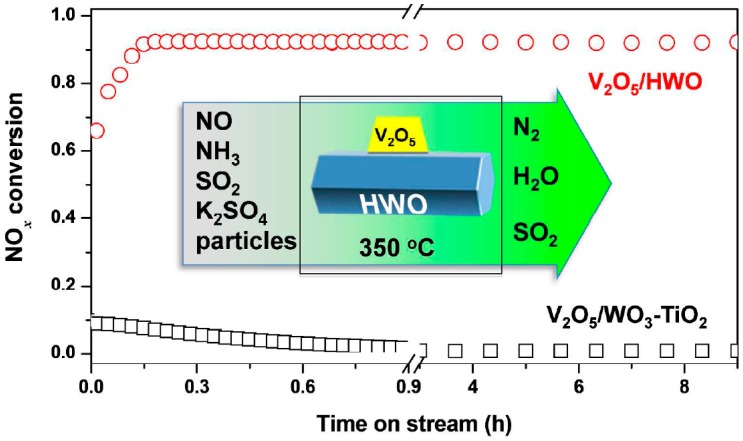
Activity evaluation of V_2_O_5_/HWO catalyst and V_2_O_5_/WO_3_-TiO_2_ catalyst at 350 °C. Reproduced with permission [53]. Copyright 2015, American Chemical Society.

In addition, the actual application of high-temperature exhaust gas may be in some alkaline environment, and the catalyst may suffer alkali poisoning. Chen, L. et al. [50] have investigated the effects that alkali metals and alkaline earth metals may have on catalysts. It was found that the degree of poisoning of the catalyst increased with the increase of alkalinity, and the N_2_O conversion rate decreased with the increase of alkaline content. The toxic element occupies the non-atomic pore site of the V_2_O_5_ [010] plane, so that the Brønsted acid and V^5+^=O sites are blocked. In alkali metals such as Na, K will reduce the stability of Brønsted acid sites, reduce the absorption of ammonia, and reduce the amount of surface chemically adsorbed oxygen. By contrast, the effect of alkaline earth metals such as Ca and Mg on the reducibility of V is not obvious. Hu, W. et al. [51] have prepared V-Ce(SO_4_)_2_/Ti catalysts, utilizing the high oxygen storage ability and strong redox ability of Ce, and sulfate SO_4_^2−^ that can increase surface acidity to obtain better performance by synergizing Ce with SO_4_^2−^. Ce introduces a high proportion of surface-active oxygen to increase the redox capacity of the catalyst. The abundant NO^+^ and NO^3-^ on the surface form a redox cycle. The catalyst has better resistance to SO_2_, H_2_O, and alkali metal than the vanadium-based commercial catalyst. Gao et al. [52] used sulfided zirconia as a catalyst support and added Ce:V = 1:1 to obtain a CeVSZ(x) catalyst. The support provides too many acidic sites to react with the alkali metal and delay the acidity reduction. V partially replaces Ce to form CeVO_4_, avoiding CeO_2_ from being converted into Ce_2_(SO_4_)_3_ to make it permanently inactivated. The CeVSZ(x) catalyst can maintain activity higher than 97% in the atmosphere of SO_2_ and K coexistence, and there is no significant decrease in activity within 400 min. Huang, Z. et al. [53] prepared a hexagonal structure of WO_3_ (HWO) as a support to load V_2_O_5_. The corner-sharing WO_6_ octahedra form hexagonal tunnels oriented along the C-axis with space group P6/mmm, and the external surface of HWO allows V_2_O_5_ to be highly dispersed. The tunnels have relatively smooth surfaces and suitable sizes for alkalis’ trapping specifically. Alkalis are readily inserted into tunnels by ion exchange reaction, so HWO has excellent resistance to alkali metal erosion. Figure 11 shows the catalytic activity evaluation of the V_2_O_5_/HWO catalyst and the V_2_O_5_/WO_3_-TiO_2_ catalyst at 350 °C under a gas containing NO, NH_3_, SO_2_ and alkali metal particles. It can be seen that the activity of V_2_O_5_/HWO is much higher than that of V_2_O_5_/WO_3_-TiO_2_ catalysts under the coexistence of SO_2_ and alkali metal.

## 5. Conclusions and Perspective

The vanadium-based catalyst is the most mature catalyst, and has a high catalytic activity at medium temperatures. However, considering the thermal instability of vanadium, the low-temperature catalytic demand, and severe working environment in industrial production, the improvement of vanadium-based catalysts has become a research hotspot. The most common improvement method is in the modification of vanadium-based catalysts. Four major systems of cerium, iron, copper and manganese are widely used in this field. Cerium has excellent ability to store and release oxygen. The redox cycle is realized by Ce^3+^/Ce^4+^ electron pairs, which provide oxygen vacancy and active sites and enhance the activity of catalysts. CeVO_4_ stabilizes the surface V^5+^ species, accelerates redox cycle and NO_2_ production, and helps to spur fast SCR. The cooperative effect between Ce and V leads to the enhancement of SCR activity. The presence of Ce^3+^/Ce^4+^ increases surface chemisorbed oxygen and provide more acid sites. CeVO_4_ promotes the formation of Brønsted acid sites while VO_x_ and CeO_2_ provide Lewis acid sites on the surface. In addition, the Lewis acid sites may be converted to the Brønsted acid sites at high temperature SCR reactions. However, Ce is prone to generate Ce_2_(SO_4_)_3_ with sulfate to cause permanent deactivation of the catalyst. At low temperatures, the unrivaled, high-activity of manganese-based oxides and copper-based oxides can significantly broaden the temperature window of vanadium-based catalysts, though they have poor low-temperature erosion resistance. The redox cycle (V^4+^ + Mn^4+^ ↔ V^5+^ + Mn^3+^) reduces the energy required for electron transfer between Mn and V active sites, and promotes the adsorption and activation of NH_3_ and NO. Mn^3+^ is conducive to NH_3_-SCR reactions because it induces the formation of more surface chemisorbed oxygen. By introducing copper species, strong acid sites are increased. It is worth noting that Lewis acid sites are the strong acid sites in Cu-V/TiO_2_ catalysts. Iron is cheap and nontoxic material with high hydrothermal stability. Due to the presence of Fe^3+^-O-V^5+^ linkages, FeVO_4_ incorporate a lot of surface defects which can adsorb or activate reactants. The adsorbed NH_3_ prefers to be activated by Fe^3+^ due to the rapid electron transfer. The presence of electronic inductive effect between Fe^3+^ and V^5+^ was beneficial for high deNO_x_ efficiency and N_2_ selectivity. The VO_x_ species, the decomposition product of FeVO_4_, exhibits higher SCR activity as active component. The presence of iron can be useful by altering the electron density of the VO_x_ surface species and inducing more Lewis acid sites and Brønsted acid sites for SCR reactions.

Although the research on denitrification catalysts has attracted widespread attention, the mechanism of SCR reaction is not yet clear. Research on the resistance of SO_2_, H_2_O, and alkali metals has made some progress, but there is still a lack of research on its inhibition mechanism. With the increasing importance of environmental protection, the development of vanadium-based catalysts is a general trend. Further study is expected to realize on the catalysis mechanism of synergistic effect and the performance optimization.
